# Human paleodiet and animal utilization strategies during the Bronze Age in northwest Yunnan Province, southwest China

**DOI:** 10.1371/journal.pone.0177867

**Published:** 2017-05-22

**Authors:** Lele Ren, Xin Li, Lihong Kang, Katherine Brunson, Honggao Liu, Weimiao Dong, Haiming Li, Rui Min, Xu Liu, Guanghui Dong

**Affiliations:** 1 MOE Key Laboratory of Western China's Environmental System, College of Earth Environmental Sciences, Lanzhou University, Lanzhou, Gansu Province, China; 2 School of Public Health, Lanzhou University, Lanzhou, Gansu Province, China; 3 Yunnan Provincial Institute of Cultural Relics and Archaeology, Kunming, China; 4 Joukowsky Institute for Archaeology and the Ancient World, Brown University, Providence, Rhode Island, United States of America; 5 College of Agronomy and Biotechnology, Yunnan Agricultural University, Kunming, China; 6 Department of Cultural Heritage and Museology, Fudan University, Shanghai, China; Museo delle Civiltà, ITALY

## Abstract

Reconstructing ancient diets and the use of animals and plants augment our understanding of how humans adapted to different environments. Yunnan Province in southwest China is ecologically and environmentally diverse. During the Neolithic and Bronze Age periods, this region was occupied by a variety of local culture groups with diverse subsistence systems and material culture. In this paper, we obtained carbon (δ^13^C) and nitrogen (δ^15^N) isotopic ratios from human and faunal remains in order to reconstruct human paleodiets and strategies for animal exploitation at the Bronze Age site of Shilinggang (ca. 2500 Cal BP) in northwest Yunnan Province. The δ^13^C results for human samples from Shilinggang demonstrate that people’s diets were mainly dominated by C_3_-based foodstuffs, probably due to both direct consumption of C_3_ food and as a result of C_3_ foddering of consumed animals. Auxiliary C_4_ food signals can also be detected. High δ^15^N values indicate that meat was an important component of the diet. Analysis of faunal samples indicates that people primarily fed pigs and dogs with human food waste, while sheep/goats and cattle were foddered with other food sources. We compare stable isotope and archaeobotanical data from Shilinggang with data from other Bronze Age sites in Yunnan to explore potential regional variation in subsistence strategies. Our work suggests that people adopted different animal utilization and subsistence strategies in different parts of Yunnan during the Bronze Age period, probably as local adaptations to the highly diversified and isolated environments in the region.

## Introduction

Analyzing ancient dietary signatures and the utilization of animal and plant resources can reveal how humans choose appropriate subsistence strategies in different cultural contexts, or under different environmental constraints [e.g. 1–9]. Archaeobotanical, zooarchaeological, and stable isotopic analyses of floral and faunal remains unearthed from prehistoric sites in China provide valuable datasets for exploring these issues. For example, recent research reveals that people adopted different subsistence strategies in order to adapt to environmental variations between China’s Loess Plateau and the high altitude Tibetan Plateau [1–2, 10], and between northern and southern China [6, 11–12]. The roles of plants and animals in ancient subsistence strategies have been studied intensively in recent years [e.g. 1, 13–16], especially through stable carbon and nitrogen isotopic analysis of human and animal bone collagen [e.g. 12, 17–24]. Isotopic methods have been widely used at prehistoric archaeological sites in the Yellow River valley [e.g. 17–20], where there is a significant distribution of Neolithic and Bronze Age sites with good bone preservation. However, in south China, especially in the Yunnan-Guizhou Plateau of southwest China, the application of stable isotope analysis in archaeological research remains limited. There is great potential to use isotopic techniques to expand our understanding of ancient subsistence systems in this environmentally and culturally diverse region.

In contrast to the Yellow River and Yangtze River valleys, where Neolithic cultures were present since the early Holocene, the Neolithic in Yunnan did not begin until around 5000 BP [25]. Neolithic and subsequent Bronze Age cultures in Yunnan Province are characterized by a good deal of variation in cultural features between micro-regions [25, 26]. The better studied Bronze Age cultures (ca. 3100–2000 BP) can be divided into four main groups, including the Dian culture type centered in the Dianchi Lake area (near Kunming), the Erhai culture type located around Erhai Lake, the Dianbian culture type in the mountainous western frontier of Yunnan, and the Honghe culture type in the Honghe (Red River) valley [26]. Although archaeologists assume that subsistence strategies were a crucial factor in the differentiation between these culture types [25], the similarities and differences in how groups exploited natural resources in various parts of Yunnan remain enigmatic.

Previous archaeobotanical studies have provided insight into plant utilization strategies in Yunnan and neighboring Guizhou Provinces during the late Neolithic and Bronze Age periods [e.g. 27–29]. The identification of plant remains from domestic crops indicates that there were three distinct phases of agricultural production in the region: from 4800–3900 BP when rice was first cultivated; from 3900–3400 BP when rice was planted together with foxtail millet and broomcorn millet; and from 3400–2300 BP when there was mixed rice, millet, wheat, and barley agriculture [28]. This last phase corresponds to the Bronze Age period. However, we do not know how important these various domestic crops were in ancient human diets. Previous zooarchaeological studies indicate that people utilized a variety of livestock including dogs, pigs, sheep, goats, and cattle during the Bronze Age in Yunnan [e.g. 30–31]. How people foddered and raised these domestic animals is not well understood.

Carbon and nitrogen stable isotope analysis provides a method for reconstructing the importance of various domestic crops and wild resources in human and animal diets and for exploring how humans utilized plant and animal resources in antiquity [32–34]. For Yunnan, data on the carbon and nitrogen isotopic values of human bone collagen have been reported for only two Bronze Age sites [24, 35]. In this paper, we present isotopic data from both human and animal bones excavated from a third site, Shilinggang, which greatly increases our understanding of Bronze Age subsistence systems in the region. Shilinggang is located in the middle course of the Nujiang River in northwest Yunnan. It is classified as belonging to the Dianbian culture type. Shilinggang was the first site to be excavated in the Nujiang River Valley, which makes it a critical location for understanding human subsistence strategies in this sub-region and for expanding our understanding of the Dianbian culture. Multi-disciplinary research involving archaeologists, archaeobotanists, zooarchaeologists, and physical anthropologists has been carried out at Shilinggang since excavations began in 2013, resulting in several preliminary publications [28, 36, 37]. Here we report a new dataset from the stable carbon and nitrogen isotope analysis of 16 human bones and 58 animal bones unearthed from Shilinggang. We compare these data with the results of archaeobotanical and stable isotopic analyses from the two other published Bronze Age sites in Yunnan in order to determine the nature of human and animal paleodiets and to analyze how people raised domesticated animals in Bronze Age Yunnan. Our results indicate that the human diet at Shilinggang relied mainly on C_3_ food, and that people used a diverse set of strategies to raise livestock.

## The study site

**Fig 1:**
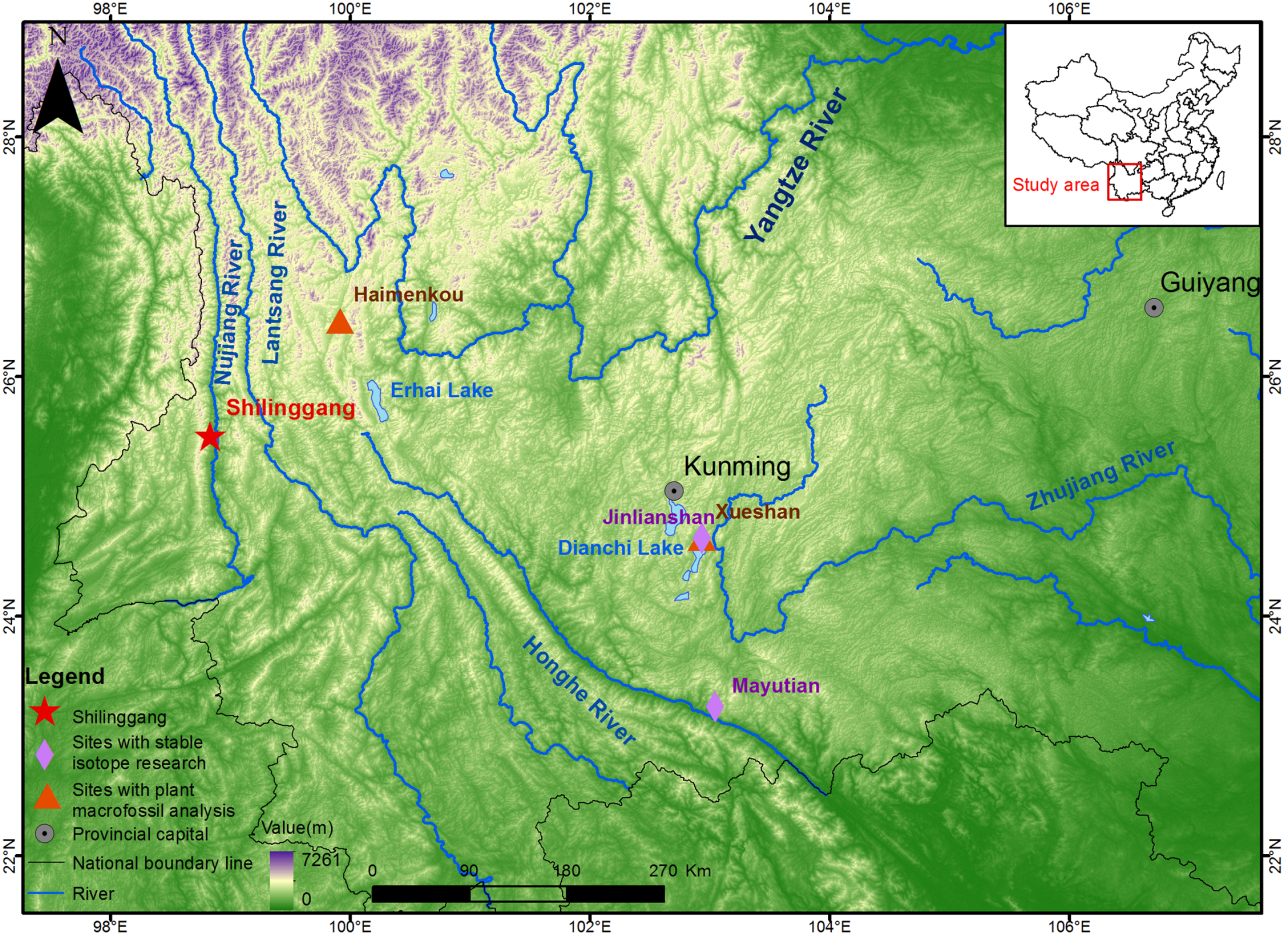
The location of Shilinggang and other sites mentioned in the text. DEM (digital elevation model) data was downloaded from Geospatial Data Cloud (http://www.gscloud.cn/), and map features in the figure were modified from Li et al. [28].

Shilinggang (N 25°38′57″, E 98°53′16″) is situated on a hill in Lushui County, Nujiang Lisu Autonomous Prefecture, northwest Yunnan, to the west of the Nujiang River (Fig 1). The site has a low-latitude plateau monsoonal climate, with an altitude of 842 m above sea level, a mean annual temperature of 20.2°C, and a mean annual precipitation level of 977.2 mm [38]. The region has high biodiversity due to its location in a low latitude area with abundant heat and light. In addition, there are numerous mountains and rivers in the area, leading to distinctively complicated topographic features that form many microclimates with intrazonal vegetation. Modern vegetation types and animal communities tend to vary with elevation [39]. Today, staple crops in the region consist of rice, maize, buckwheat, wheat, roots, and tubers, while meat is provided by livestock and occasional hunting [40]. In the past, abundant wild and domestic floral and faunal resources would have provided a wide range of options to fulfill human dietary needs.

**Fig 2:**
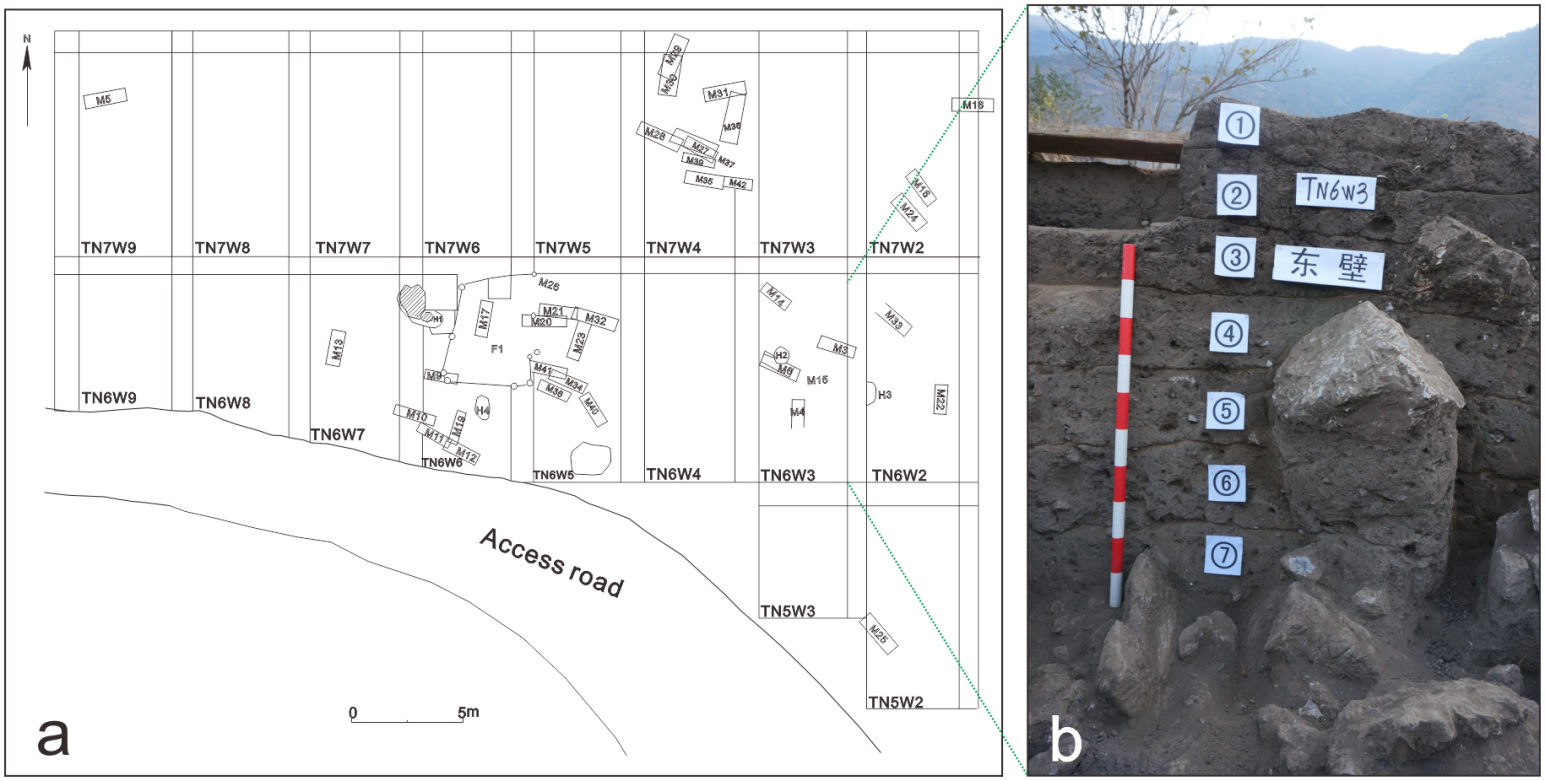
Plan map of northern Shilinggang and stratigraphic column from unit TN6W3. a) Plan map of the northern part of Shilinggang. Rectangular excavation units are labeled starting with the letter T. Features include burials (labeled with the letter “M”), pits (labeled with the letter “H”), and building foundations (labeled with the letter “F”). The features shown in Fig 2a are modified from Li et al. [28]. b) Photograph of stratigraphic profile in unit TN6W3 (center right of plan map in Fig 2a). Circles around numbers indicate stratigraphic levels.

The site of Shilinggang was first discovered and investigated in 2003 by the Nujiang Prefectural Administration of Cultural Relics. It was further investigated in 2007 by the Yunnan Provincial Institute of Cultural Relics and Archaeology and the Nujiang Prefectural Administration of Cultural Relics. The first formal excavations of the site, which were also the first archaeological excavations in the Nujiang River Valley, were conducted in 2013 and 2014 by the Yunnan Provincial Institute of Cultural Relics and Archaeology. The site has a higher elevation in the north than the south. It covers a total area of nearly 100,000 m^2^ with 2 m deep accumulations of cultural materials. The northern part of the site has thicker cultural layers that were excavated according to rectangular excavation units (Fig 2a). All of the materials analyzed in this study were excavated from the northern part of the site. Additional excavations in the central and southern parts of the site were conducted by excavating long narrow trenches. A total area of 500 m^2^ has been excavated to date.

Stratigraphic divisions separate the site into seven cultural layers from top to bottom (Fig 2b). In total, excavators uncovered 42 graves, 4 ash pits, 2 activity floors, 2 house foundations, and the remains of burnt soil. Excavated artifacts include pottery, stone tools, bronze vessels, plant remains, and large quantities of human and animal bones. Graves account for the overwhelming majority of excavated features. Skeletons in these graves are relatively complete even though some bones are poorly preserved. There are no obvious traces of groundwater flow or other transportation of bones out of the graves. We believe that remains in grave features were not significantly disturbed through taphonomic processes.

All of the tombs are rectangular with a vertical earthen shaft. Most graves are single burials while a few are joint burials containing both adults and children buried together. In terms of size, graves are relatively small (110–260 cm long, 36–68 cm wide and 10–48 cm deep) and the vast majority contain no burial goods. Only about 10% of graves contain one or two bronzes, stone tools, or ceramic objects. There is no obvious evidence from mortuary remains to indicate the existence of strict social class differences. While house foundations and ash pits are few in number, the distribution of graves among the houses and other features suggests that there were no spatial divisions between areas of the site used for artifact production, living, burial, and other activities.

Artifacts excavated from all seven cultural layers at Shilinggang are consistent with the material culture of the Spring and Autumn and Warring States periods (770–221 BC) [36]. Two charred rice seeds from the fourth cultural layer of square TN6W2 and fifth cultural layer of square TN5W3 were directly radiocarbon dated to between 2723 Cal BP and 2339 Cal BP at 2σ, confirming that the site dates to the late Bronze Age [28]. Flat land in this area of Yunnan is scarce, and there are many large canyons that cut through the landscape. It is possible that the gentle slope of Shilinggang was a preferred place to live and grow crops, which may explain the site’s deep accumulation of Bronze Age cultural materials [36].

## Material and method

### Material selection

A total of 16 human bone samples (all from graves in the northern part of Shilinggang, including 2 child samples and 14 adult samples) and 58 animal bone samples (all from cultural layers in northern Shilinggang, including one sheep (*Ovis aries*), 4 goats (*Capra hircus*), 5 samples that could be either sheep or goats (*Ovis aries/Capra hircus*), 10 cattle (*Bos* sp.), 4 dogs (*Canis familiaris*), 13 pigs (*Sus* sp.), 2 Old World monkeys (Cercopithecidae), 12 muntjacs (*Muntiacus* sp.), and 7 unidentified deer (Cervidae)) were selected for stable isotope analysis. Bones from human graves were assigned to age and sex, if possible, based on standard morphological traits [41], by Dongyue Zhao of Jilin University. Animal bones were identified by Lele Ren of Lanzhou University. All samples date to the Spring and Autumn and Warring States periods. All human and animal bones are currently stored in Yunnan Provincial Institute of Cultural Relics and Archaeology, Kunming. Tables 1 and 2 list each sample including information on species, skeletal element, sample code, sample excavation context, and bone collagen quality control indicators.

**Table 1:** Isotopic composition and quality indicators of animal samples from Shilinggang, Yunnan. Samples marked in bold italics were found to be contaminated, and were not included in further statistical analysis. Context locations are shown in Fig 2a. Among the sample context codes, TNnWn (where n is an Arabic numeral) refers to excavation unit; circles with a number inside refer to the stratigraphic layer.

Species	Skeletal element	Symmetry	Code	Sample context	%Yield	%C	%N	C/N ratio	δ^13^C (‰)	δ^15^N (‰)
Goat	Metatarsal	Left	21	TN6W2⑤	1.9	43.8	15.6	3.3	-15.4	3.3
Sheep	Humerus	Left	22	TN6W7②	19.7	41.7	14.4	3.4	-21.6	4.5
Goat	Humerus	Left	23	TN6W5⑤	16.7	43.1	15.1	3.3	-9.6	3.6
Sheep/goats	Humerus	Left	24	TN6W5⑥	2.2	42.8	15.3	3.3	-22.9	4.9
***Goat***	***Tibia***	***Left***	***26***	***TN6W2⑤***	***0*.*9***	***40*.*8***	***14*.*1***	***3*.*4***	***-21*.*9***	***6*.*9***
Goat	Radius	Left	27	TN6W2⑤	1.7	42.2	14.8	3.3	-21.6	7.3
Sheep/goats	PhalanxI	Left	28	TN7W4③	13.6	43.5	15.5	3.3	-22.4	5.0
Sheep/goats	Mandible	Left	29	TN6W5④	4.8	43.2	15.4	3.3	-10.3	3.0
***Goat***	***Tibia***	***Left***	***30***	***TN5W2②***	***0*.*8***	***43*.*0***	***14*.*9***	***3*.*4***	***-23*.*9***	***5*.*4***
Sheep/goats	Tibia	Left	31	TN6W6	5.0	43.7	15.4	3.3	-23.0	4.0
Goat	PhalanxI	Left	32	TN6W2③	4.1	43.6	15.2	3.3	-21.5	6.1
Sheep/goats	PhalanxI	Left	33	TN6W5 M20	1.0	38.3	13.6	3.3	-22.6	7.1
Cattle	Metacarpal	Left	34	TN7W4③	7.6	42.7	15.0	3.3	-23.6	5.6
***Cattle***	***Metacarpal***	***Left***	***35***	***TN6W6⑤***	***0*.*6***	***40*.*6***	***13*.*2***	***3*.*6***	***-12*.*7***	***4*.*6***
Cattle	Radius	Left	36	TN6W2⑤	2.6	42.0	14.8	3.3	-22.9	6.2
Cattle	Metacarpal	Left	37	TN6W6	3.7	42.2	14.8	3.3	-17.9	4.3
Cattle	Metatarsal	Left	38	TN6W3⑤	2.9	43.5	15.5	3.3	-13.2	4.5
Cattle	Metacarpal	Left	39	TN7W4②	2.4	44.1	15.7	3.3	-14.2	4.6
Cattle	Metacarpal	Left	40	TN6W7①	2.9	40.9	14.1	3.4	-22.8	3.7
***Cattle***	***PhalanxI***	***Left***	***41***	***TN6W5⑥***	***0*.*7***	***41*.*4***	***14*.*3***	***3*.*4***	***-12*.*3***	***4*.*5***
Cattle	Metatarsal	Left	42	TN6W7②	6.3	43.4	15.3	3.3	-17.6	4.9
Cattle	Metacarpal	Left	44	TN6W3③	8.7	44.2	15.7	3.3	-13.5	5.1
Cattle	PhalanxI	Left	45	TN6W3⑥	6.1	42.7	15.1	3.3	-16.9	4.6
Cattle	PhalanxII	Left	46	TN7W4⑤	4.9	42.8	15.1	3.3	-11.2	3.4
***Cattle***	***PhalanxI***	***Left***	***47***	***TN5W2②***	***0*.*6***	***43*.*0***	***15*.*0***	***3*.*3***	***-13*.*0***	***5*.*4***
***Cattle***	***PhalanxI***	***Left***	***48***	***TN6W5④***	***0*.*5***	***44*.*0***	***15*.*6***	***3*.*3***	***-15*.*0***	***4*.*9***
Muntjac	Metacarpal	Left	62	TN6W5③	5.0	42.7	15.6	3.2	-21.3	5.6
Muntjac	Radius	Left	63	TN7W4⑤	4.7	44.0	15.5	3.3	-25.9	4.4
Muntjac	Radius	Left	64	TN6W6	1.4	41.1	13.3	3.6	-22.1	5.4
Muntjac	Humerus	Left	65	TN6W6	5.3	42.2	14.7	3.4	-21.7	5.4
Muntjac	Metacarpal	Left	66	TN6W5⑥	7.7	43.4	15.4	3.3	-21.3	5.4
Muntjac	Radius	Left	67	TN6W7②	2.0	42.9	15.1	3.3	-22.4	4.8
Muntjac	Humerus	Left	68	TN6W7	4.6	43.8	15.6	3.3	-21.8	6.4
Muntjac	Tibia	Left	69	TN6W5⑤	8.3	43.1	15.3	3.3	-22.6	5.1
Muntjac	Metatarsal	Left	71	TN7W4②	2.5	44.3	15.6	3.3	-21.9	4.3
Muntjac	Tibia	Left	72	TN6W3⑤	6.3	43.9	15.7	3.3	-21.4	5.5
Muntjac	Tibia	Left	73	TN6W7①	2.1	43.2	15.3	3.3	-21.9	5.7
***Muntjac***	***Metacarpal***	***Left***	***75***	***TN7W4①***	***0*.*8***	***42*.*8***	***14*.*8***	***3*.*4***	***-21*.*7***	***5*.*5***
Muntjac	Radius	Left	76	TN6W6⑥	3.2	43.4	15.4	3.3	-22.5	7.0
Deer	Metacarpal	Left	77	TN6W3⑥	9.8	43.6	15.7	3.2	-24.5	4.1
Deer	Metatarsal	Left	78	TN6W2⑤	3.4	42.1	14.9	3.3	-22.6	5.8
Deer	Metatarsal	Left	79	TN6W3③	1.9	32.8	11.7	3.3	-24.2	4.9
Deer	Metatarsal	Left	80	TN6W5②	6.3	43.6	15.5	3.3	-23.4	4.2
Deer	Metacarpal	Left	81	TN6W2⑦	1.4	43.3	15.2	3.3	-23.6	4.7
Deer	Metacarpal	Left	82	TN6W2③	8.1	43.9	15.8	3.2	-19.9	5.1
Deer	PhalanxII	Left	83	TN6W6	5.0	43.7	15.6	3.3	-23.1	4.8
Old World monkey	Femur	Left	84	TN6W2③	6.0	44.2	15.3	3.4	-18.9	8.0
Old World monkey	Ulna	Left	85	TN6W7①	2.1	44.0	15.5	3.3	-18.8	10.6
Dog	Radius	Left	13	TN7W2③	3.8	43.0	15.3	3.3	-20.5	8.0
Dog	Femur	Left	14	TN6W6	4.5	43.3	15.5	3.3	-14.8	7.4
Dog	Radius	Left	17	TN5W2②	3.2	43.3	15.5	3.3	-18.9	8.4
Dog	Humerus	Left	18	TN7W4②	6.8	42.4	15.1	3.3	-18.9	7.4
Pig	Ulna	Left	49	TN7W2①	5.6	43.0	15.1	3.3	-20.1	8.2
Pig	Humerus	Left	50	TN5W2②	3.3	43.1	14.8	3.4	-20.1	8.8
Pig	PhalanxI	Left	51	TN6W6⑥	2.4	43.5	15.2	3.3	-22.7	3.6
Pig	Scapula	Left	52	TN6W5③	1.7	42.8	14.9	3.4	-15.9	6.6
Pig	Metatarsal	Left	53	TN6W3⑥	2.5	42.9	15.7	3.2	-18.5	6.3
Pig	Radius	Left	54	TN6W2③	4.8	41.9	15.1	3.2	-20.4	8.8
Pig	Humerus	Left	55	TN6W3②	1.9	41.4	14.7	3.3	-20.3	8.7
Pig	Ulna	Left	56	TN6W6⑤	4.4	38.9	13.7	3.3	-20.8	5.8
Pig	Radius	Left	57	TN6W7②	1.4	42.8	15.5	3.2	-20.6	5.0
Pig	Ulna	Left	58	TN6W7①	3.4	42.0	15.1	3.2	-19.5	8.3
Pig	Radius	Left	59	TN7W4⑤	1.4	40.1	14.7	3.2	-21.0	4.8
Pig	Humerus	Left	60	TN6W3⑤	3.5	41.5	15.0	3.2	-17.5	6.7
Pig	PhalanxII	Left	61	TN6W6	2.0	41.0	15.1	3.2	-20.8	4.8

**Table 2:** Isotopic composition and quality indicators of human samples from Shilinggang, Yunnan. The samples marked in bold italics were found to be contaminated, and were not included in further statistical analysis. Context locations are shown in Fig 2a. Among the sample context codes, TNnWn (where n is an Arabic numeral) refers to excavation unit; Mn (where n is an Arabic numeral) refers to the grave number. “Ind.” stands for indeterminate sex.

Age (years)	Sex	Skeletal element	Symmetry	Code	Sample context	%Yield	%C	%N	C/N ratio	δ^13^C (‰)	δ^15^N (‰)
Adult	Female	PhalanxI	Left	126	TN6W3 M3	3.1	43.9	15.7	3.3	-19.0	9.8
40+	Female	Femur	Left	128	TN7W2 M24	7.2	43.9	15.7	3.3	-18.9	7.5
40–45	Female	Tibia	Left	129	TN6W6 M17	2.4	43.9	15.6	3.3	-19.4	9.6
30–35	Female	Radius	Left	133	TN6W6 M11	6.5	43.7	15.5	3.3	-19.1	9.9
***Adult***	***Female***	***Fibula***	***Left***	***136***	***TN6W3 M14***	***0*.*4***	***41*.*5***	***14*.*0***	***3*.*5***	***-19*.*3***	***9*.*8***
Adult	Female	Femur	Left	137	TN7W2 M18	1.6	43.1	14.8	3.4	-19.5	10.5
Adult	Male	Radius	Left	130	TN6W5 M21	2.9	44.2	15.6	3.3	-16.3	9.3
***Adult***	***Male***	***Radius***	***Left***	***135***	***TN7W9 M5***	***0*.*5***	***43*.*9***	***14*.*8***	***3*.*5***	***-16*.*7***	***8*.*9***
***40–50***	***Male***	***Fibula***	***Left***	***140***	***M2***	***0*.*3***	***42*.*9***	***14*.*3***	***3*.*5***	***-17*.*7***	***9*.*9***
35–0	Male	Humerus	Left	145	TN6W3 M15	1.5	41.8	14.8	3.3	-19.2	10.2
Adult	Ind.	Radius	Left	131	TN6W6 M10	1.4	43.9	15.2	3.4	-18.6	10.4
Adult	Ind.	Radius	Left	132	M1	1.1	43.0	15.0	3.3	-18.5	10.3
40+	Ind.	Fibula	Left	138	TN6W6 M9	8.6	43.2	15.4	3.3	-18.9	10.5
Adult	Ind.	Radius	Left	141	TN6W5 M20	1.1	44.4	15.0	3.4	-18.2	8.5
***Adult***	***Ind*.**	***Fibula***	***Left***	***142***	***TN6W6 M12***	***0*.*6***	***44*.*5***	***14*.*8***	***3*.*5***	***-18*.*8***	***10*.*3***
Adult	Ind.	Tibia	Left	143	TN6W6 M17	1.2	44.7	15.5	3.4	-19.1	10.2
25+	Ind.	Metapodial	Left	144	TN6W6 M19	1.5	45.0	15.9	3.3	-19.2	9.4
Adult	Ind.	Ulna	Left	146	TN9W7 M7	1.7	43.2	15.1	3.3	-18.8	11.1
5±	Ind.	Radius	Left	127	TN7W2 M16	2.8	43.2	15.0	3.4	-19.1	11.3
***8–9***	***Ind.***	***Fibula***	***Left***	***134***	***TN6W3 M6***	***0*.*7***	***44*.*4***	***15*.*2***	***3*.*4***	***-19*.*4***	***10*.*5***
4–5	Ind.	Ulna	Left	139	TN6W2 M22	2.3	44.8	15.7	3.3	-18.6	11.1

### Collagen extraction

Bone collagen was extracted from the human and animal samples following the protocol outlined by Jay and Richards [42], with some modification described in Ambrose et al. [43]. For all human and animal bones, a dense bone fragment of approximately 3 g was removed from each whole bone sample. We used a large 3 g sample size, which is more than the usual 200 mg used in similar studies, in order to ensure that enough bone collagen could be extracted from each sample since we predicted that the acidic soil at Shilinggang would lead to poor collagen preservation. An electric grinder was used to grind the outer and inner surface of the bone to remove surface contamination before further processing. After cleaning, the bone fragments were demineralized by soaking in 0.5 M HCl at 4°C, refreshing the solution every two days, until the bone became soft and no bubbles were emitted. The residue was washed repeatedly with deionized water to neutral pH, and then rinsed in 0.125 M NaOH for 20 h at 4°C to remove soil humic acids. Samples were washed again with deionized water to neutral pH, dissolved in 0.001 M HCl at 70°C, and put in a dry oven for two days to gelatinize. The gelatinized solution was filtered when hot and then freeze-dried using a Labconco* FreeZone Lypholizer for 48 h to retain the collagen. The final collagen was saved and weighed for later analysis.

### Measurement of organic carbon and organic nitrogen content and stable isotope ratios

The organic C and N content and the stable isotope ratios of the collagen samples were measured using a multi-flow isotope-rationing mass-spectrometer (Isoprime 100 IRMS) combined with an elemental analyzer (Vario PYRO cube) in the Archaeological Stable Isotope Lab, Department of Scientific History and Archaeometry, University of the Chinese Academy of Sciences, Beijing, China. Before the measurement, sulfanilamide was added as a reference standard to calibrate the C and N content. Caffeine (IAEA-600, δ^13^C: -27.8±0.0‰, δ^15^N: 1.0±0.2‰), sucrose (IAEA-cH-6, δ^13^C: -10.5±0.1‰) and (NH_4_)_2_SO_4_ (IAEA-N-2, δ^15^N: 20.3±0.2‰) were used to normalize both N_2_ (AIR as standard) and CO_2_ (VPDB as standard). During the testing process, a sulfanilamide sample and a laboratory standard sample (CAAS, δ^13^C: -14.7±0.1‰, δ^15^N: 7.0±0.1‰) were inserted into the collagen list every ten samples for real-time monitoring and calibration. The analytical precision on the results of samples repeated in triplicate was better than 0.2‰ for both carbon and nitrogen isotopic ratios.

### Data analysis

All statistical analyses were carried out with IBM SPSS Statistics 19 for Windows. Hierarchical cluster analysis was used to analyze differences in collagen δ^13^C and δ^15^N values among samples (S1 Text, S1 Fig). The resulting dendrograms (constructed using average linkage between groups) were used to separate samples into groups with significantly different isotopic signatures that reflect groups of individuals with different diets or foddering strategies (S2 and S3 Figs). Hierarchical cluster analysis is a preferred statistical method over other tests such as K-means because it auto-generates clusters of similar data points without requiring the analyst to pre-establish the number of output groups. We also tested the dietary groups identified through hierarchical cluster analysis using the K-means method and found that these groups are apparent in either method. A One-sample Kolmogorov-Smirnov Test was used to test whether the data was normally distributed, and if they were not, a non-parametric Mann-Whitney U test was used. The significance level was set at p < 0.05.

## Results and discussion

### Bone collagen preservation and contamination

The chemical composition and biological characteristics of bone are influenced and changed by temperature, pH levels, humidity, and microorganisms in the burial environment, processes which together are referred to as bone diagenesis [44]. A prerequisite of dietary reconstruction is that bone must retain its original chemical and isotopic composition even after a long period of deposition. In Tables 1 and 2, we include three reliable measurements of collagen preservation quality, including the collagen yield (% yield), the content of C (% C) and N (% N), and the atomic ratio of C/N. Out of the total 86 samples, 74 samples with collagen representing more than 1% of the original bone mass were used for analysis (mean collagen yield = 4.1%, SD = 3.1). The other 12 samples with less than 1% collagen yield were discarded because it is impossible to eliminate potential contaminants in samples with very low collagen yield [45–46]. In addition, the percentages of C (mean = 42.8±0.2%) and N (mean = 15.1±0.1%) in the 74 samples included in the analysis are in close proximity to those of modern bone collagen (C: 41%, N: 15%) [45], indicating that the ancient collagen is well preserved. The atomic C/N of these samples (mean = 3.3), ranged from 3.2 to 3.6, which falls within the 2.9–3.6 range considered to be a quality indicator for excellent collagen preservation [47]. The 16 human and 58 animal samples which meet the above criteria can be considered well preserved for stable isotope analysis.

### Animal stable isotope data and paleodietary analysis at Shilinggang

The isotopic composition of ingested food is reflected in body tissues [48]. Stable isotope ratios in bone collagen and apatite generally indicate the isotopic composition of an individual’s diet over a period of years [49, 50]. The average carbon stable isotopic value (δ^13^C) of an organism can be related to C_3_ vs. C_4_ ecosystem dominance [51] or marine vs. terrestrial food webs [52]. C_3_-plants have more negative δ^13^C values (-27.1±2.0‰) compared to C_4_-plants (-13.1±1.2‰) because of different mechanisms of CO_2_ fixation during photosynthesis [53]. In China, foxtail millet, a common C_4_ crop found at archaeological sites, has a much less negative δ^13^C value compared to C_3_ crops such as rice or other wild vegetation [54, 55]. Macrobotanical remains and phytoliths of both foxtail millet and rice have been identified at Shilinggang [28]. Trophic level also influences stable isotope ratios. Bone collagen δ^13^C values are enriched about 0–2‰ between prey animals and their predators [32]. A significant enrichment in δ^15^N also occurs between an organism’s diet and its body tissues, leading to δ^15^N values 3–5‰ higher in the body than in the average diet [56], or up to 6‰ in some cases [57]. However, nitrogen isotopic ratios are affected by many other factors as well, such as manuring, breastfeeding, aridity, salinity, and nutritional stress [58–63].

**Fig 3:**
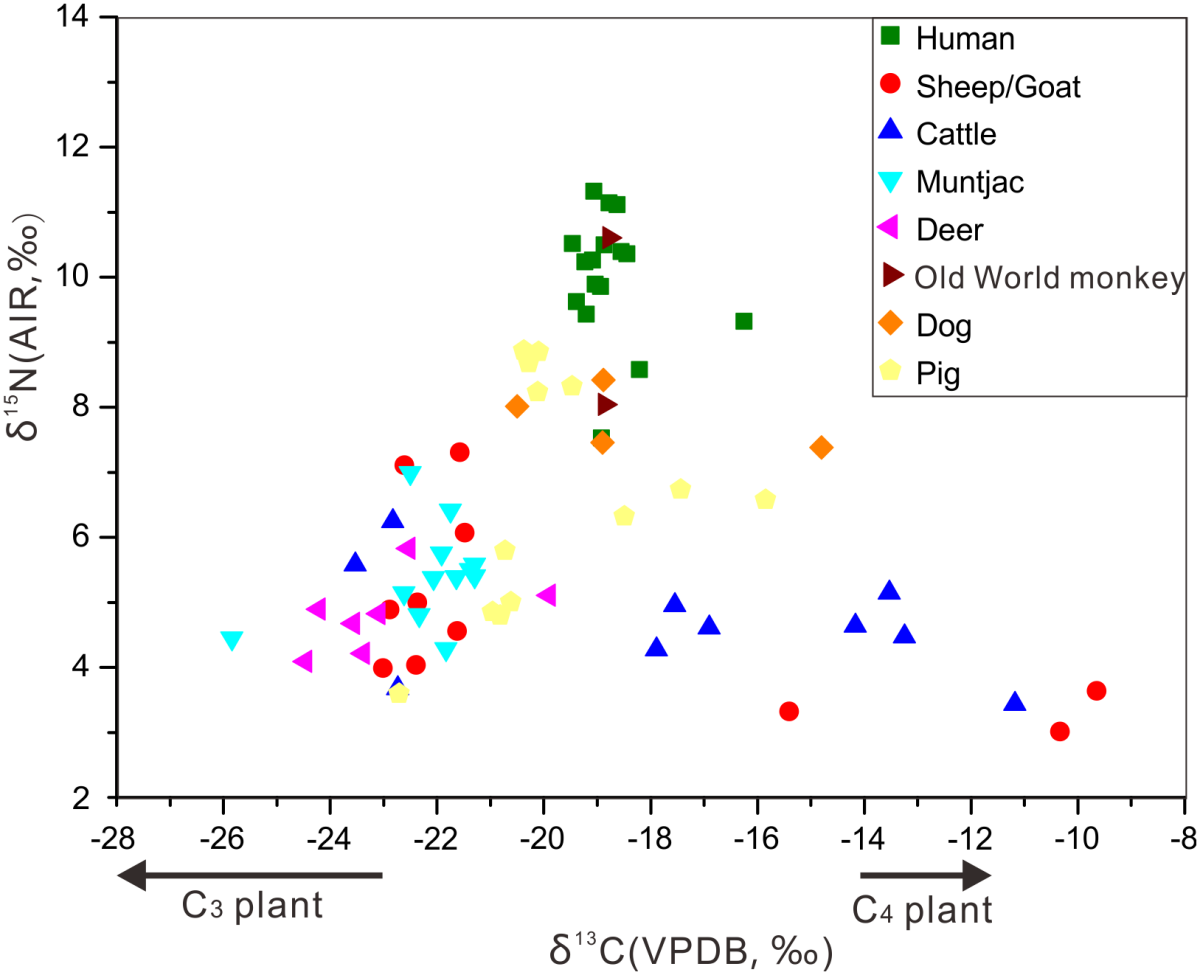
Scatter diagram of human and animal collagen carbon and nitrogen values from Shilinggang. Analytical error is so small that it is contained within the symbols.

The δ^13^C and δ^15^N values from the Shilinggang bone samples (Tables 1 and 2) are presented in the scatter diagram in Fig 3. Stable isotope analysis of the animal bones provides a baseline for understanding the local food web [64]. For convenience in further discussion, the animal data were classified into the following groups: wild herbivores (muntjac and deer); omnivores (dogs and pigs); domesticated herbivores (sheep/goats and cattle); and primates (monkeys).

The δ^13^C values of wild herbivores (n = 19, 12 muntjacs and 7 deer) range from -25.9‰ to -19.9‰ (mean = -22.5±1.4‰) and the δ^15^N values range from 4.1‰ to 7.0‰ (mean = 5.2±1.0‰), indicating a C_3_ plant-based herbivorous diet. The narrow range of δ^13^C values suggests that these wild herbivores ate similar types of plants, and is consistent with the natural ecological background of C_3_ plant-dominated vegetation in the region [39].

For the omnivore group (n = 17, 4 dogs and 13 pigs), all dogs were identified as fully domesticated in accordance with their bone morphological characteristics while pigs were identified as either domestic or wild. The δ^13^C values of dogs range between -20.5‰ and -14.8‰ (mean = -18.3±2.4‰). It appears that three of the dogs were fed predominantly with C_3_ food, which possibly includes rice, root and tuber crops, and/or animal protein based on C_3_ food. The one other dog with the highest δ^13^C value (-14.8‰) consumed a mixed C_3_ and C_4_ diet, suggesting that its diet likely contained millet and/or animal protein from animals that ate millet. The δ^15^N values of the dog bones have a narrow range from 7.4‰ to 8.4‰ (mean = 7.8±0.5‰). The offset of nitrogen stable isotope between wild herbivores and dogs is 2.6‰, which approaches the range of the N isotopic enrichment (3‰-5‰) of one trophic level [32, 65], indicating that Shilinggang dogs consumed animal protein in quantities possibly through association with humans [17]. Dogs likely lived in close proximity to their human owners. These dietary signatures may reflect intentional provisioning of dogs, but could also indicate that dogs ate human waste and food scraps available around the site.

**Fig 4:**
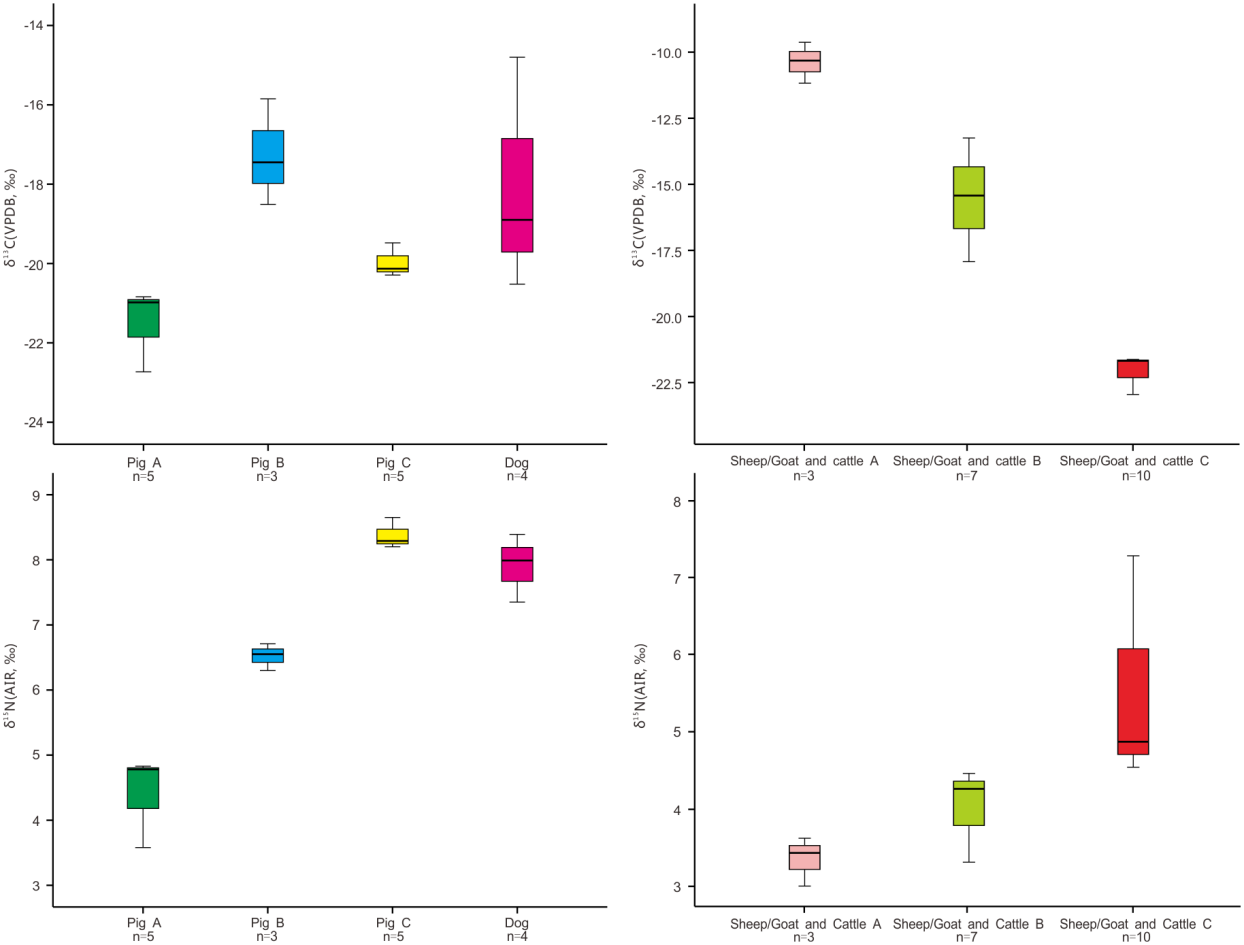
Isotopic value distributions for domesticated animals showing groups with different husbandry strategies.

The 13 pig samples can be divided into three groups (labeled as pig A, pig B and pig C, respectively in Fig 4) based on differences in δ^13^C and δ^15^N values revealed through Hierarchical Cluster analysis (S2 Fig). The pig A group includes 5 samples with the lowest δ^13^C values (mean = -21.2±0.9‰) and δ^15^N values (mean = 4.8±0.8‰). These values are similar to those of the wild herbivores. These individuals likely represent wild boars consuming C_3_-based plants and little animal protein. The morphological characteristics of the bones that provided samples in the pig A group are also more similar to wild boar than domestic pigs. However, we can’t rule out the possibility that individuals in the pig A group could be domesticated, but free-range. Three pig samples with the highest δ^13^C values (mean = -17.3±1.3‰) and middle-range δ^15^N values (mean = 6.5±0.2‰) fall into the pig B group. These animals had a mixed C_3_ and C_4_ diet. C_4_ plant species comprise a minority of terrestrial plants (less than 4%) and are maladapted to most shady temperate environments [66], such as the environment of western Yunnan. Pollen cores from Lake Erhai indicate that the most common plants in Yunnan during the late Bronze Age were C_3_ plants, especially *Pinus*, and deciduous arboreal trees [67]. Although the distribution of wild C_4_ plants in China is not well known, we assume that most of the natural flora in the region has low δ^13^C values. Given that the only known C_4_ plant at Shilinggang is foxtail millet, we argue that the C_4_ signal in the pig B group represents the consumption of domestic millet, its by-products, and/or animals that fed on millet.

The remaining five pig samples correspond to the pig C group, with a middle-range mean δ^13^C value of -20.1±0.4‰ and the highest mean δ^15^N value of 8.6±0.3‰. These animals appear to have been at a similar trophic level to dogs and humans (Fig 3). It is likely that the pigs in group C were domesticated and, like most dogs, scavenged on mainly C_3_ food scraps or were provisioned by people with C_3_ foods. The diet also included plenty of animal protein. Stable carbon and nitrogen isotope analysis has been applied to track pig domestication at many other archaeological sites in China [18, 68, 69]. Our results indicate that δ^13^C values are also useful for distinguishing between remains of domesticated pigs (pig groups B and C) and wild boars (pig group A) at Shilinggang.

Hierarchical Cluster Analysis revealed that domesticated herbivores (10 sheep/goats and 10 cattle) could also be divided into three groups according to their δ^13^C values (S3 Fig and Fig 4). Based on previous isotopic studies of ancient Chinese fauna [18–19, 55], these groups represent three distinct dietary characteristics: group A represents individuals with diets of mainly C_4_ plants (> -12‰), group B represents individuals with diets of mixed C_3_ and C_4_ plants (-18‰ to -12‰), and group C represents individuals with diets of predominantly C_3_ plants (< -18‰). Group A is composed of two sheep/goats samples and one cattle sample with the highest δ^13^C values (mean = -10.4±0.8‰) and lowest δ^15^N values (mean = 3.4±0.3‰). These animals largely fed on C_4_ plants, very likely millet by-products. One sheep/goats sample and six cattle samples in group B are identified as mixed C_3_ and C_4_ eaters, with mean δ^13^C and δ^15^N values of -15.5±1.9‰ and 4.5±0.6‰, respectively. The δ^13^C values likely represent a mixed C_4_ signal from consumption of millet and a C_3_ signal from consumption of rice and/or wild forage [28, 67]. Group C contains seven sheep/goats samples and three cattle samples with a mean δ^13^C value of -22.5±0.7‰ and a mean δ^15^N value of 5.4±1.2‰. This group consumed predominantly C_3_ plants, likely rice and/or the natural vegetation surrounding the site [28, 67]. The mean of both δ^13^C values (Mann-Whitney test: U = 91.500, Z = -0.161, P = 0.872) and δ^15^N values (Mann-Whitney test: U = 84.000, Z = -0.505, P = 0.614) between group C and the wild herbivores are not significantly different, demonstrating that the domestic sheep/goats and cattle in group C were likely free-range animals that ate wild C_3_ vegetation [67]. Another possibility is that some of these animals actually represent wild bovids native to the region such as gaur, takin, serow, or goral, rather than domestic bovids [70]. The diverse isotopic signatures for the domesticated herbivores, representing three distinct dietary groups, may reflect three different husbandry strategies used by people at Shilinggang. The subtropical natural environment in northwestern Yunnan may have provided abundant year-round forage for most herd animals. However, people also provided supplemental fodder to some sheep, goats, and cattle using millet agricultural products. A few individuals were foddered very intensively with millet or may have grazed in millet fields.

Two Old World monkey samples (Cercopithecidae) have δ^13^C and δ^15^N values of -18.9‰ and 8.0‰ (#84) and -18.8‰ and 10.6‰ (#85), respectively, showing that they are characterized by a predominantly C_3_ omnivorous diet with high-levels of protein. The stable isotope values are consistent with the diet of most monkeys in the region, which is composed of C_3_ plants (including tender leaves, sprouts, flowers, fruit, and seeds), insects which feed on C_3_ plants, and bird eggs [71–72]. The δ^15^N value of sample #84 approaches those in omnivorous nonhuman primates, which suggests that its diet included insects or vertebrate fauna [73–74]. Sample #85 has approximately 3‰ higher δ^15^N indicating an offset of one trophic level from sample #84. This sample was from an unfused distal ulna, indicating that it came from a sub-adult animal. The higher trophic level may be due to the consumption of maternal milk [e.g. 73–75], with this sample representing a homogenized signal from the first few years of life due to slow bone turnover rates.

### Human stable isotope data and paleodietary analysis at Shilinggang

**Fig 5:**
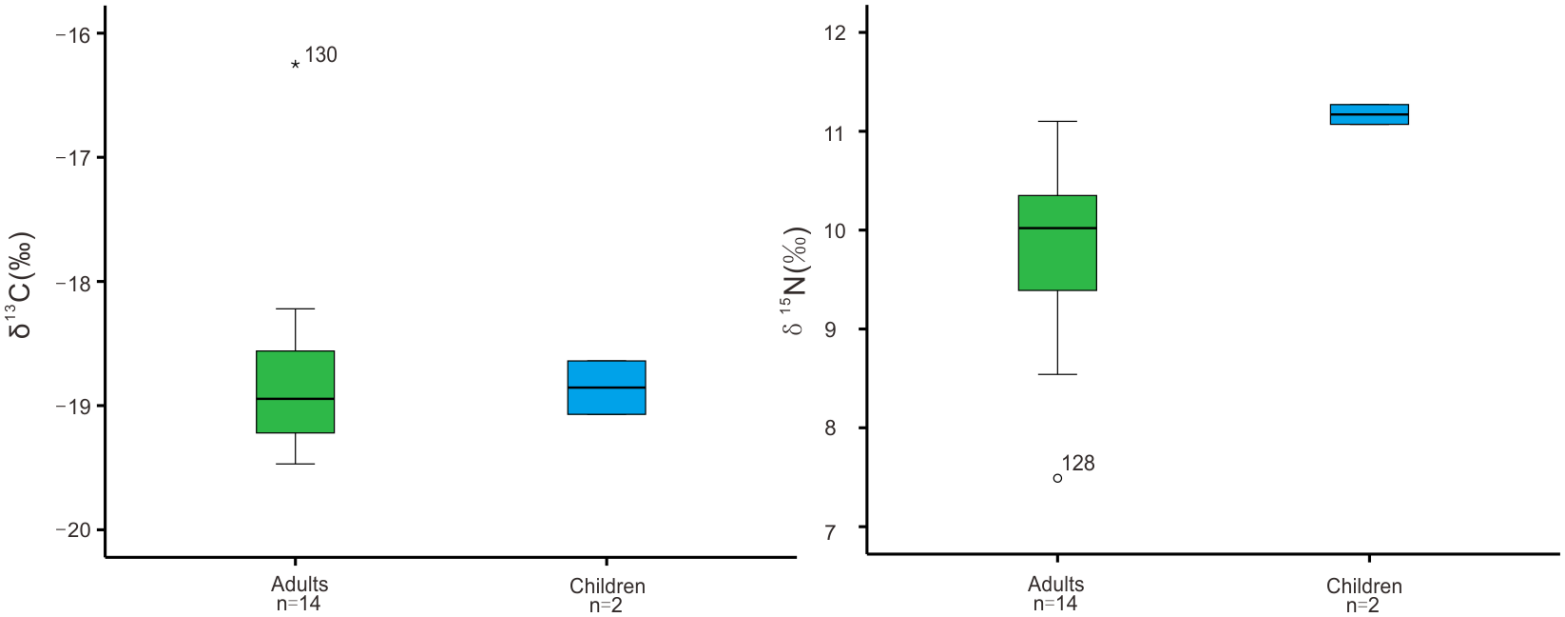
Comparison of human isotopic signatures for adult vs. child samples.

The carbon and nitrogen data for the 16 human samples from Shilinggang are listed in Tables 2 and 3, and plotted in Figs 3 and 5. The δ^13^C values for the human bones range from -19.5‰ to -16.3‰ (mean = -18.8±0.8‰), and the δ^15^N values vary from 7.5‰ to 11.3‰ (mean = 10.0±1.0‰). In accordance with previous studies [18–19, 55], human δ^13^C signatures can be divided into three main dietary groups: mainly C_3_ consumers (< -18‰); mixed C_3_ and C_4_ consumers (-18‰ to -12‰); and highly C_4_ dependent consumers (> -12‰). The population of Shilinggang appears to have been predominantly C_3_ consumers. It is likely that people’s diet consisted primarily of rice [28], as well as roots, tubers, fruits, and/or animals fed on a C_3_ diet. However, there is one individual (#130) with a δ^13^C of -16.3‰ who consumed a small amount of C_4_ foodstuffs, likely foxtail millet and/or animals fed on millet [28]. The mean δ^15^N value for humans is 4.8‰ higher than that of wild-herbivores and 3.1‰ higher than that of omnivores, suggesting that people consumed abundant animal protein. People at Shilinggang might have consumed both wild animals available in the region or domesticated livestock kept near the site. Consumption of freshwater fish could also lead to a high δ^15^N value [76], which may be a complementary reason for the high δ^15^N values in the human samples. However, very few fish bones have been found in the excavation of Shilinggang. Zooarchaeological analysis of animal remains from Shilinggang indicates that domesticates were the most common animals present, making up about 60% of identified specimens. However, a diverse array of wild fauna including several types of deer, boar, badger, porcupine, bear, monkeys, birds, and fish were also exploited in small numbers. Together the isotopic, paleobotanical, and zooarchaeological data indicate that people at Bronze Age Shilinggang developed a broad-spectrum economic strategy that included agriculture (planting rice, millet, roots, and tubers), animal husbandry (raising pigs, dogs, sheep/goats, and cattle), hunting wild animals, and freshwater fishing.

**Table 3:** Summary of human and animal isotopic data. Adults include samples from individuals over 25 years old. Children include two bone samples from individuals under 25 years old. SD: Standard deviation.

Species	Sample number (n)	δ^13^C(‰)			δ^15^N(‰)		
		Average	SD (2σ)	Range	Average	SD (2σ)	Range
All human	16	-18.8	0.8	-19.5--16.3	10.0	1.0	7.5–11.3
Children	2	-18.9	0.3	-19.1--18.5	11.1	0.1	11.1–11.3
Adult	14	-18.8	0.8	-19.5--16.3	9.8	0.9	7.5–11.1
Sheep/goats	10	-19.1	5.3	-23.03--9.63	4.9	1.5	3.0–7.28
Cattle	10	-17.4	4.5	-23.55--11.17	4.7	0.8	3.43–6.22
Dog	4	-18.3	2.4	-20.52--14.8	7.8	0.5	7.35–8.39
Pig	13	-19.9	1.7	-22.73--15.85	6.6	1.8	3.58–8.84
Old World Monkey	2	-18.8	0.1	-18.87--18.79	9.3	1.8	8.01–10.56
Wild herbivore (Muntjac and Deer)	19	-22.5	1.4	-25.87--19.92	5.2	0.7	4.07–6.96

We divided human samples into two groups based on age (adult vs. child) to see if there are demographic differences in dietary signatures. Two bone samples from children were estimated to be around 4 to 5 years old at the time of death, which provide some early childhood dietary information for the Shilinggang population. The child samples have δ^13^C values ranging from -19.1‰ to -18.5‰ (mean = -18.9±0.2‰) and δ^15^N values ranging from 11.1‰ to 11.3‰ (mean = 11.2±0.1‰). The other 14 adult samples have δ^13^C values ranging from -19.5‰ to -16.3‰ (mean = -18.8±0.2‰) and are not significantly different from the two samples representing childhood diets (Mann-Whitney test: U = 13.000, Z = -0.159, P = 0.933). This indicates that people at Shilinggang consumed C_3_ foodstuffs during both childhood and adulthood. The weak signal of difference between adults and children (0.1‰) may simply result from the small sample size for children. Alternately, if children consumed more C_3_ plant food after being weaned this could also weaken the signal. The δ^15^N values for the 14 adults range from 7.5‰ to 11.1‰ (mean = 9.8±0.2‰) and display a significant difference from the two samples representing children’s diets (Mann-Whitney test, U = 1.000, Z = -2.064, P = 0.033). The samples representing children’s diets have enriched δ^15^N values by 1.4‰, which could likely be attributed to the consumption of breast milk [e.g. 74–75], but could also be affected by the small sample size.

As Fig 3 and Table 2 show, there are two outliers (#128 and #130) among the human samples. Sample #128 has a δ^13^C value of -18.9‰ and a δ^15^N value of 7.5‰, which shows that this individual had a predominately C_3_ diet similar to most other people at the site. However, the δ^15^N value for this individual is 2.5‰ less than the average value of all human samples (10.0±1.0‰), indicating that this person consumed less animal protein. There are several possible explanations. This person may have had low social status (e.g. a slave, prisoner of war, criminal, etc.), and her diet may have included less protein than other members of the population [e.g. 35, 77–78]. The diet difference could also represent personal preference or a cultural choice. Perhaps she did not like eating animal products or was a migrant to Shilinggang from other regions where people ate less meat. Sample #130 has a δ^15^N value of 9.3‰, which is similar to other individuals at the site, but the δ^13^C value of -16.3‰ is relatively high, suggesting a mixed C_3_ and C_4_ diet. This person was not only consuming rice, roots, and/or tubers and wild plants (C_3_ plants), but also some foxtail millet (C_4_ plant). The mixed paleodietary signature from this sample is accordant with archaeobotanical evidence showing that people made use of diverse plant food resources at Shilingang [28].

### Human diets and animal utilization strategies during the Bronze Age in Yunnan

In addition to our current study, two additional stable isotope analyses of human remains from Yunnan have been published for the Jinlianshan cemetery (2700–2300 cal BP) and Mayutian site (2500–2400 cal BP) (Fig 1) [24, 35]. No stable isotopic data for faunal remains have been reported from these two sites. Therefore, our analysis of human and animal bone samples from Shilinggang provides an important isotopic baseline for understanding human and animal dietary structures in Yunnan during the Bronze Age. Stable isotope results from our current study as well as those from Jinlianshan and Mayutian indicate that people may have had varied diets in different regions of Yunnan. For example, at Jinlianshan bone collagen δ^13^C values have a limited range from -19.3‰ to -18.2‰ (mean = -18.8±0.4‰, n = 9) [35, Table 4]. This is quite different from the large range of bone collagen δ^13^C values from -19.5‰ to -16.3‰ (mean = -18.8±0.8‰, n = 16) that we found at Shilinggang. Despite the greater range of δ^13^C values at Shilinggang, the mean δ^13^C values (Mann-Whitney test: U = 68.000, Z = -0.226, P = 0.846) and δ^15^N values (Mann-Whitney test: U = 59.000, Z = -0.736, P = 0.487) between Shilinggang and Jinlianshan have no significant difference. When compared to Jinlianshan and Shilinggang, the population at the site of Mayutian appears to have had a very different diet made up of both C_3_ and C_4_ resources, but statistical comparison is not possible because isotope data from Mayutian is derived from apatite rather than collagen [24]. More data is still needed, but the current evidence suggests that Bronze Age groups at Shilinggang and in the Jinlianshan cemetery consumed C_3_-based foods, while those at Mayutian mainly had a mixed diet of both C_3_ and C_4_ resources [24, 35]. The high proportion of C_3_ plants in people’s diets in this part of China may also indicate that in addition to growing domestic C_3_ plants such as rice, people also took advantage of the wild plant resources available locally.

**Table 4:** Human collagen carbon and nitrogen isotope results from sites in Yunnan.

Site	Approximate date (Cal BP)	Location	δ^13^C (‰)				δ^15^N (‰)				Reference
			N	Mean	SD(2σ)	Range	N	Mean	SD(2σ)	Range	
Jinlianshan	2500–2200	Kunming	9	-18.8	0.4	1.2	9	9.8	0.9	2.6	35
Shilinggang	2700–2300	Nujiang	16	-18.8	1.0	3.2	16	10.0	1.0	3.8	28; This study

We do not yet have a complete understanding of the regional variation in agricultural practices and animal husbandry among Yunnan’s different culture groups. At Shilinggang, rice and foxtail millet are the only domestic crops that have been identified in the paleobotanical record [28]. At other Bronze Age sites such as Haimenkou and Xueshan (Fig 1), rice, wheat, barley, foxtail millet, and broomcorn millet have all been identified [79–80]. This indicates that people in Bronze Age Yunnan cultivated a wide variety of domestic crops. Because carbon and nitrogen isotopic data from animal bone collagen have not been reported from Jinlianshan and Mayutian, estimating the contribution of animal foods to ancient human diets at these two sites is impossible. Our comparison of nitrogen isotopic values for human and animal bone collagen at Shilinggang indicates that people consumed a large amount of animal protein during the late Bronze Age, and that it came primarily from C_3_ fed domestic or wild animals. Our analysis also identified dietary groups such as pig group A and sheep/goats and cattle group C that may represent the additional exploitation of wild boar and wild bovids.

Bronze Age animal exploitation in Yunnan seems to have focused on domestic animal husbandry supplemented by hunting of wild animals. Zooarchaeological analyses of other archaeological sites in the region have identified similar domestic livestock and wild animals as we have found at Shilinggang [30–31]. However, the ways that humans raised domestic animals in this region have not been discussed in detail. The isotopic signatures for faunal remains from Shilinggang suggest that humans might have raised livestock using multiple herd management strategies. We found that the δ^13^C values for domesticated sheep/goats and cattle suggest they had three different kinds of diets containing variable amounts of C_4_ plants (Fig 4). Pigs can also be split into three similar dietary groups that may reflect wild vs. domestic populations and different amounts of millet by-products in the diet.

The animal dietary signatures at Shilinggang may also reflect seasonal changes in herding practices or differences in the types of grazing present in different micro-regions. The landscape of the Nujiang River Valley changes dramatically with increasing altitude. During the Bronze Age, rain-fed millet crops may have been preferentially cultivated in the highlands, while rice may have been planted in the lowlands [28]. Accordingly, people might have herded animals in different ways at different points along the slopes or at different times of year, feeding animals with the by-products of the plants grown at various elevations. Transhumance may explain some of the variation in carbon and nitrogen isotopic values between the sheep/goats and cattle groups at Shilinggang, and future isotopic studies of the region should consider this possibility in more detail.

Although more data from additional sites is needed, we propose that regional cultures in different areas of Yunnan developed subsistence strategies according to local environmental conditions. Yunnan has highly diverse and isolated ecological environments split by numerous large rivers, such as the Nujiang, Lancang, and Jinsha Rivers and the Hengduan Mountains (Fig 1) [81]. Mountainous terrain with limited flat land for planting crops likely promoted the development of independent cultural systems within subregions, and the exploitation of abundant wild plant and animal resources. These geographic barriers may have also hindered cultural and technological exchange between regions [81]. Research in other parts of the world has shown that geographic variation results in diversification of subsistence strategies [e.g. 82–86]. Our research provides a starting point for examining the relationships between environmental diversity and subsistence in Yunnan. In the coming years, additional isotopic, paleobotanic, and zooarchaeological studies will further clarify the subsistence systems and cultural variation within this unique region.

## Conclusion

Stable isotope data from Shilinggang reveals the diverse subsistence strategies and animal husbandry techniques that were used during the late Bronze Age in southwest China. Dogs and pigs had diets that consisted of mainly C_3_ foodstuffs and were high in animal protein. Their diets were similar to those of humans at the site, likely resulting from the consumption of human food waste. However, pigs can be divided into three separate groups, including pig A representing wild boar and pig B and C two types of domestic individuals based on their isotopic signatures. Sheep/goats and cattle specimens can also be divided into three separate dietary groups. These dietary differences may reveal the presence of wild vs. domestic populations, but also indicate that some herd animals were more intensively foddered with millet by-products. Humans generally consumed a large amount of C_3_ foodstuffs and a large amount of animal protein, supplemented with C_4_ foods. Together the data suggest that people at Shilinggang developed diversified economic strategies that focused on agriculture and domestic animal husbandry, supplemented by collecting wild plants, hunting, and fishing. We propose that the subsistence systems used by different cultural groups in the various regions of Yunnan may have been adapted to local environmental conditions, resulting in considerable spatial differentiation of Bronze Age cultures in this region.

## Supporting information

S1 Text Hierarchical cluster analysis. (DOCX)

S1 Fig Operation process and parameter setting of hierarchical cluster analysis. (TIF)

S2 Fig Hierarchical cluster analysis of collagen d^15^N and d^13^C values from pig samples. The three main groups are labeled A-C. The dendrogram was constructed using average linkage (between groups).
(TIF)

S3 Fig Hierarchical cluster analysis of collagen d^15^N and d^13^C values from sheep/goats and cattle samples. The three main groups are labeled A-C. The dendrogram was constructed using average linkage (between groups).
(TIF)
