# Supplementary material for: Human paleodiet and animal utilization strategies during the Bronze Age in northwest Yunnan Province, southwest China
Source: PLoS One. 2017 May 22;12(5):e0177867. doi: 10.1371/journal.pone.0177867 (PMC5439680; doi:10.1371/journal.pone.0177867)
Supplement: S1 Text — (DOCX) [file pone.0177867.s001.docx]

**S1 Text. Hierarchical cluster analysis.**

We divided stable carbon and nitrogen isotopic data of pigs and domesticated herbivores including sheep/goats and cattle into three groups, respectively, using hierarchical cluster analysis. All data were processed with hierarchical cluster analysis included in the software IBM SPSS Statistics 19 for windows (S1 Fig). The metric for cluster analysis that we used was the Euclidean distance, which is the most common distance measure used in published studies. The criterion that we used to group the data was average linkage clustering between groups, with the threshold set according to the system default. The analysis results were presented as dendrograms (S2 Fig for pigs and S3 Fig for sheep/goats and cattle).
