# Supplementary figures and images for: Human paleodiet and animal utilization strategies during the Bronze Age in northwest Yunnan Province, southwest China
Source: PLoS One. 2017 May 22;12(5):e0177867. doi: 10.1371/journal.pone.0177867 (PMC5439680; doi:10.1371/journal.pone.0177867)

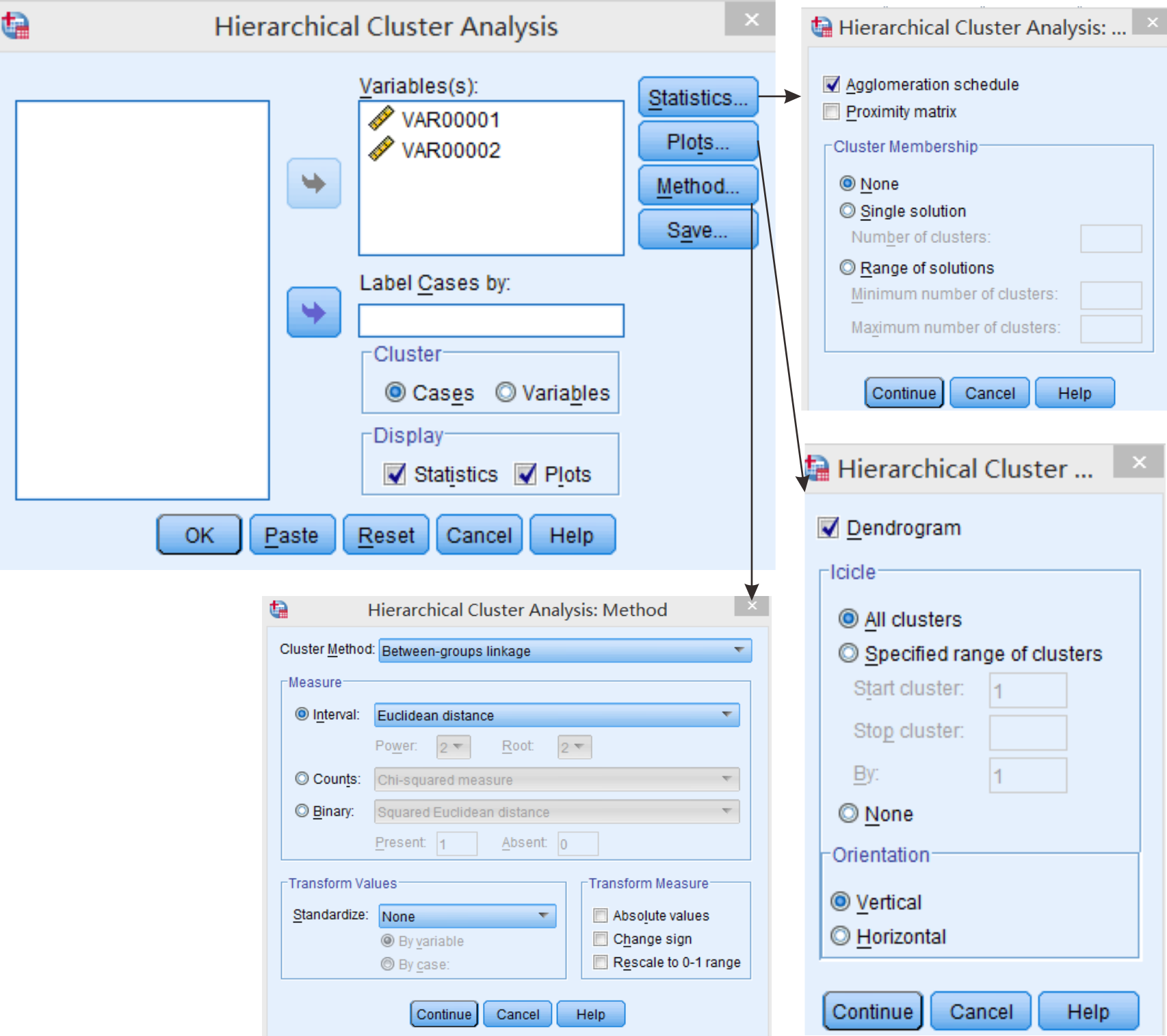

Supplement: S1 Fig — (TIF) [file pone.0177867.s002.tif]

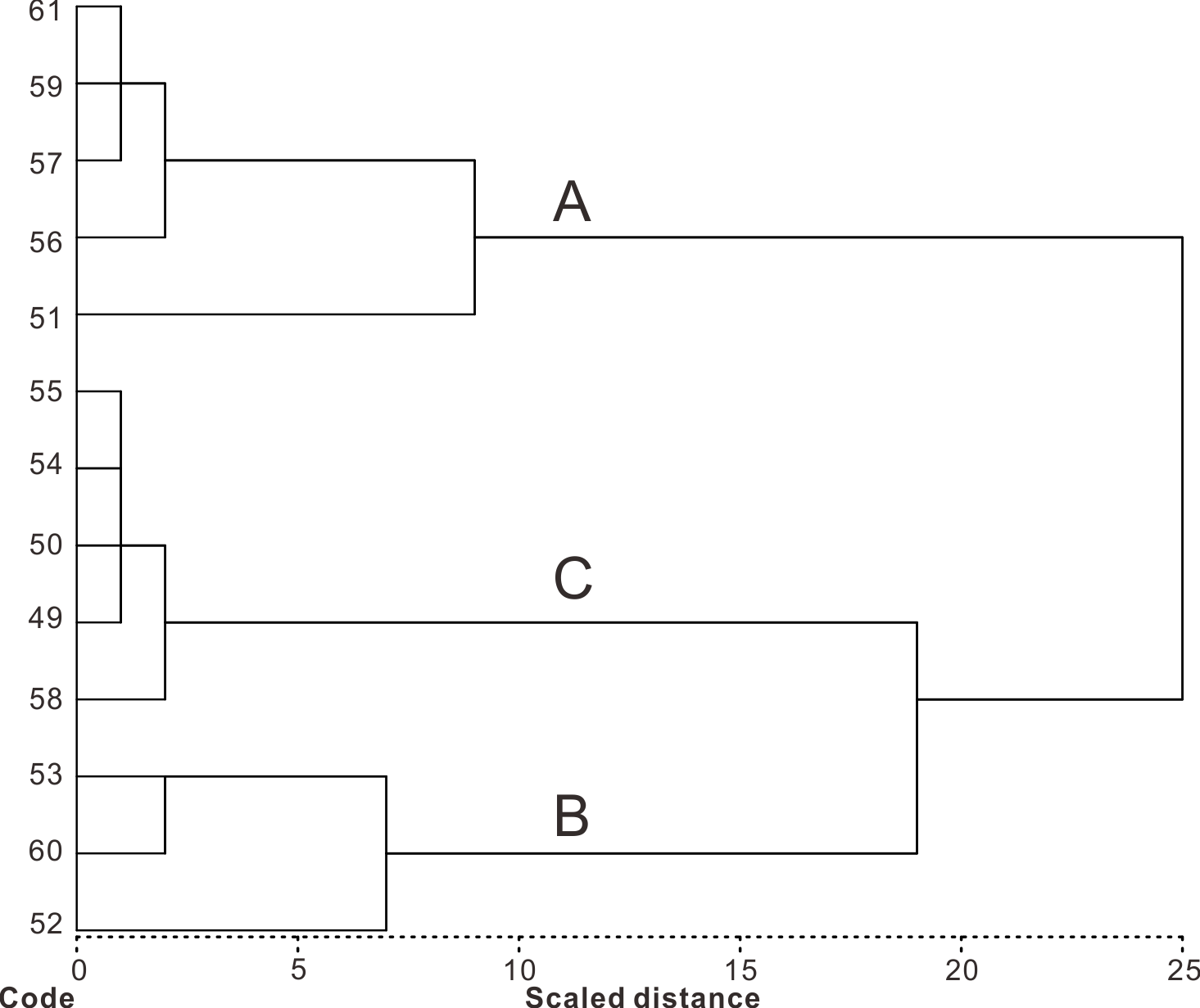

Supplement: S2 Fig — The three main groups are labeled A-C. The dendrogram was constructed using average linkage (between groups). (TIF) [file pone.0177867.s003.tif]

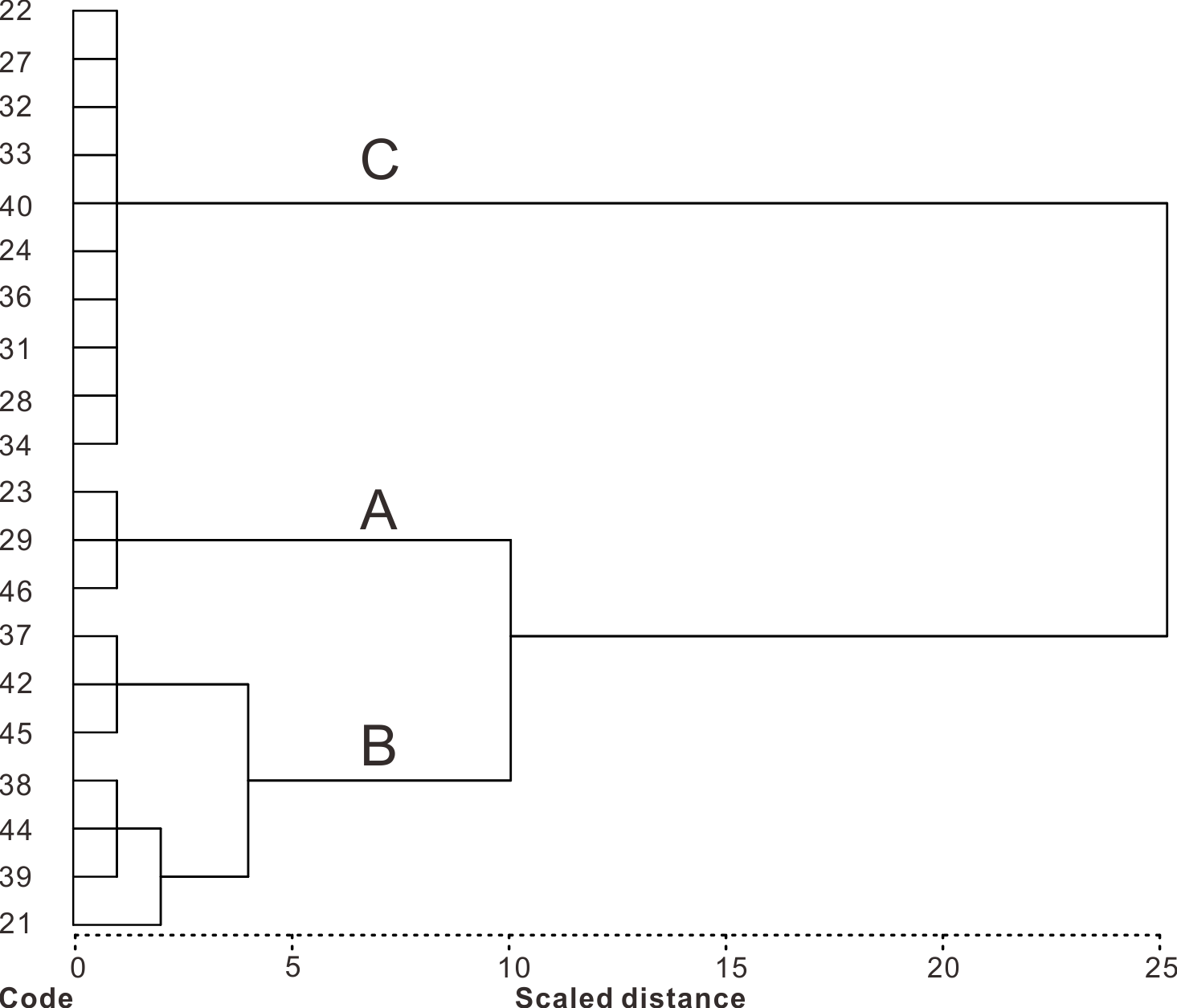

Supplement: S3 Fig — The three main groups are labeled A-C. The dendrogram was constructed using average linkage (between groups). (TIF) [file pone.0177867.s004.tif]
